# Analysing the causes of chronic cough: relation to diesel exhaust, ozone, nitrogen oxides, sulphur oxides and other environmental factors

**DOI:** 10.1186/1745-6673-1-6

**Published:** 2006-05-18

**Authors:** Beatrix Groneberg-Kloft, Thomas Kraus, Anke van Mark, Ulrich Wagner, Axel Fischer

**Affiliations:** 1 Division of Allergy Research, Charité – Universitätsmedizin Berlin, Free University and Humboldt-University, 13353 Berlin, Germany; 2 Institute of Occupational Medicine, University School of Medicine, RWTH Aachen, 52074 Aachen, Germany; 3 Institute of Occupational Medicine, University Lübeck, D-23538 Lübeck, Germany; 4 Department of Medicine, Pulmonary and Critical Care Division, Philipps-University, D-35043 Marburg, Germany

## Abstract

Air pollution remains a leading cause of many respiratory diseases including chronic cough. Although episodes of incidental, dramatic air pollution are relatively rare, current levels of exposure of pollutants in industrialized and developing countries such as total articles, diesel exhaust particles and common cigarette smoke may be responsible for the development of chronic cough both in children and adults. The present study analyses the effects of common environmental factors as potential causes of chronic cough. Different PubMed-based researches were performed that related the term cough to various environmental factors. There is some evidence that chronic inhalation of diesel can lead to the development of cough. For long-term exposure to nitrogen dioxide (NO2), children were found to exhibit increased incidences of chronic cough and decreased lung function parameters. Although a number of studies did not show that outdoor pollution directly causes the development of asthma, they have demonstrated that high levels pollutants and their interaction with sunlight produce ozone (O3) and that repeated exposure to it can lead to chronic cough. In summary, next to the well-known air pollutants which also include particulate matter and sulphur dioxide, a number of other indoor and outdoor pollutants have been demonstrated to cause chronic cough and therefore, environmental factors have to be taken into account as potential initiators of both adult and pediatric chronic cough.

## Introduction

Coughing and mucus secretion are coordinated neuronal reflexes that protect the respiratory tract from noxious exogenous substances under physiological conditions. However, within chronic exposure to noxious substances such as tobacco smoke, urban dust, or occupational factors [1-3], the originally protective mechanisms may lead to a states of chronic distress with hypersecretion and chronic coughing [4-10]. The neurophysiology of the cough reflex and its relation to bronchoconstriction and different forms of adult and pediatric asthma is very complex [11]. However, there is little doubt that chronic cough can be related to the exposure to different environmental air pollutants. Amongst them, pollutants such as diesel exhaust, ozone, nitrogen and sulphur dioxide have all been suggested to participate as main causes or co-factors in the development of chronic cough [12].

These suggestions do not only base on epidemiological and clinical observations, but also on the neurophysiological and -anatomical understanding of the cough reflex. In this respect it is generally accepted, that airway pollutant-caused airway irritation leading to chronic cough displays a complex phenomenon involving a variety of reflex mechanisms [13-19].

Neurophysiologically, a subgroup of rapidly adapting receptors (RARs) among the three major types of vagal sensory receptors is suggested to act as "cough receptors". Next to these RARs, a further effect by bronchopulmonary C-fibers on the cough reflex has been suggested, and there are data indicating that i.e. ozone, one of the main environmental air pollutants, exerts an influence on vagal-sensory innervation [20-24]. Also, transient receptor potential vanilloid-1 seems to play a role in the mediation of the cough reflext [25,26] and airway nerves and their mediators in general are likely to play an important role in the general pathology of cough and airway inflammation [27-33].

**Figure 1:**
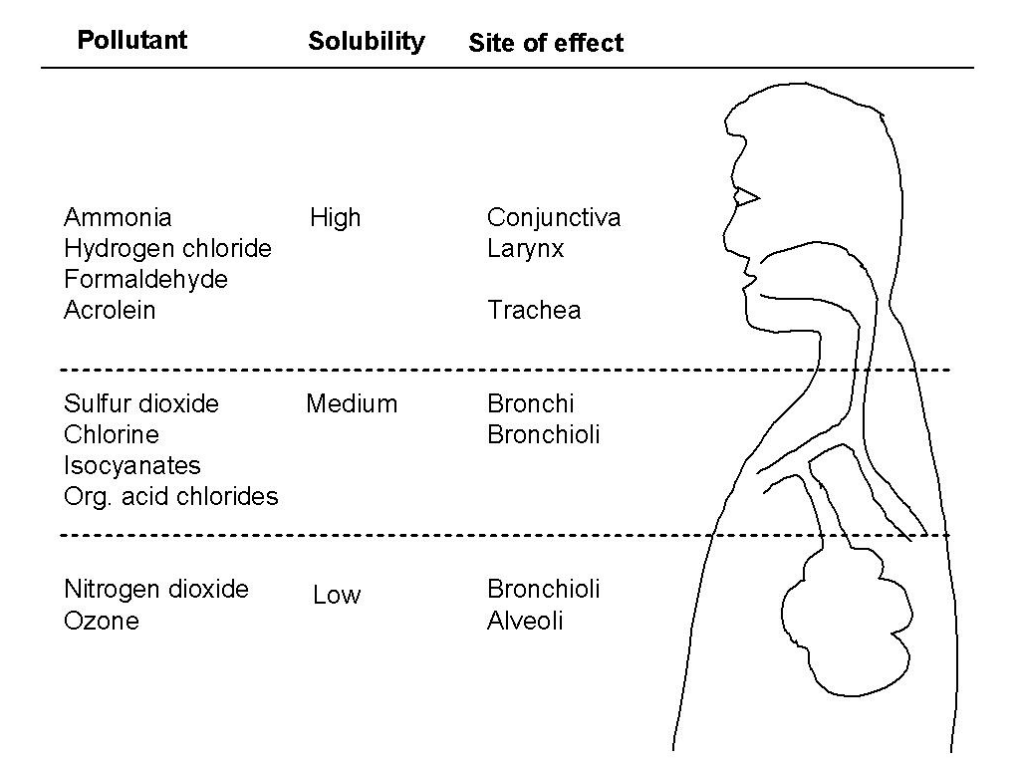
**Deposition of environmental polluatants in the respiratory tract**. (modified from [11]).

In the light of the clinical, epidemiological and experimental data which point to a major role of environmental pollutants as co-factors for the development and progression of chronic cough, the present study analysed the data available on the association between environmental pollutants and chronic cough on the basis of a large amount of existing recent literature reviews and original articles [12,13,34-55]. Figure 1 illustrates the deposition of some environmental pollutants related to cough in the respiratory tract.

## Material and methods

### Methods

A PubMed research was performed using terms such as "cough", "environmental", and various environmental factors and publication types (date: 2006-03-03). Articles were screened for their contents and relevant data was analysed.

## Results and discussion

### Frequency of research related to cough and environmental factors

**Figure 2:**
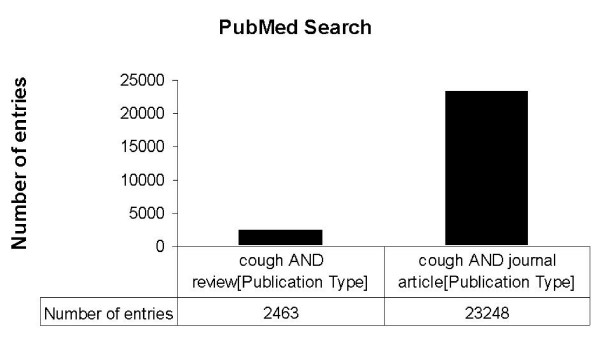
PubMed search for the terms "cough" and publication types.

**Figure 3:**
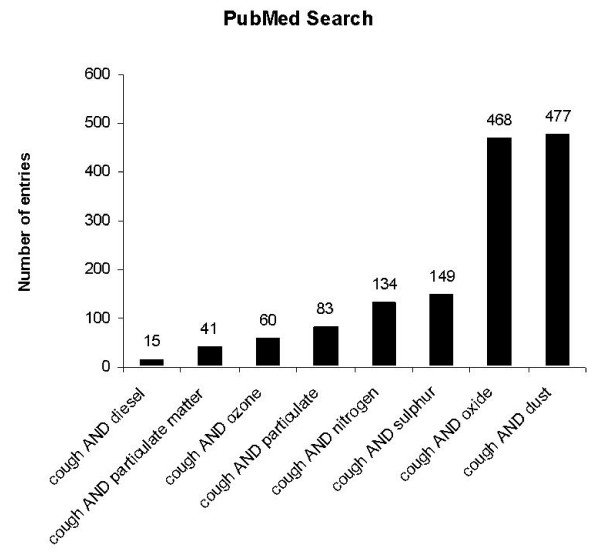
PubMed search for the terms "cough" and different environmental factors.

**Figure 4:**
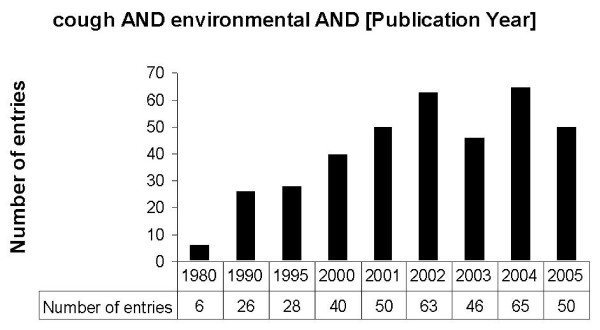
Frequency of studies related to cough and environmental factors as assessed by a PubMed search for the terms "cough" and "environmental" and different publication dates.

For the terms „cough”and journal article as publication type 23248 entries were registrated in the PubMed while 2463 review articles containing the term cough were found (Fig. 2). To analyse specific articles related to environmental medicine, the search was narrowed and different terms related to environmental factors were encluded (Fig. 3). To analyse the frequency of scientific studies related to environmental factors and cough, different publication dates were analysed in in general, an increasing frequency was found beginning i.e. in the year 1980 with 6 articles and increasing from 1995 with 28 articles to a number of 65 articles in the year 2004 (Fig. 4).

### Diesel exhaust

The term "diesel" was only found in a low frequency in studies related to cough (Fig. 3). Next to health effects caused by exposure to tobacco smoke, there has been an increasing attention to the effects of diesel emissions in the past years [12]. Both on the epidemiological and the experimental field, a variety of studies demonstrated a relation between diesel exhaust and respiratory diseases such as chronic cough, asthma, chronic bronchitis or cancer. Diesel exhausts consists of a variety of components: Next to products arising from the incomplete combustion (carbon monoxide, nitrogen oxides, hydrocarbons including partially oxidized forms such as ketones, aldehydes, phenols, and sulphur compounds), diesel exhaust contains substances deriving from the complete air and carbon combustion such as nitrogen, carbon dioxide and water. In comparison to gasoline engine exhausts, diesel emissions contain a far greater amount of nitrogen oxides and aldehydes but less carbon monoxide. Next to these components, diesel exhaust also includes submicron particles and fine particles below 10 μm which can cause a variety of respiratory effects.

Chronic effects of exposure to diesel emission have been assessed by several studies and the prevalence of chronic cough varied from 29 to 37 % among smokers and between 8 to 16 % in nonsmokers. The risk ratio (observed/expected) for diesel emission exposed individuals varied between 1.2 and 2.3 and there was a consistent finding for an increased risk of chronic cough among the studies [56-59]. In general, the non-malignant respiratory effects of diesel exhaust exposure can be summarized as an increased prevalence of chronic cough and also of phlegm production and of dyspnea. Also, the FEV_1 _of diesel exposed individuals can show a significant decrease. However, the diesel emissions can not be incriminated as indirect or direct causes due to confounding by other exposures such as tobacco smoke and for the most part of the data, no exposure-response relationships are present.

### Nitrogen dioxide

Nitrogen dioxide (NO2) is an oxidizing free radical which can initiate a number of destructive pathways in the human body [12,60]. NO2 plays an important role in atmospheric pollution and might be a major cause of human respiratory problems such as chronic cough in urban areas [61]. Numerous studies have addressed a relationship between NO2 and chronic cough (Fig. 3).

In 1985, primary schoolchildren and their mothers were surveyed in Hong Kong to study the possible relationship of nitrogen dioxide (NO2)-related environmental air pollution to respiratory illnesses [62]. Using personal samplers to measure NO2, the study also assessed the major sources of NO2 in the indoor environment and examined if an increased NO2 concentration is positively associated with respiratory symptoms. The levels of NO2 among the examined 319 mothers were increased by 21% if there was dust exposure found at the mothers' workplace. There was an increase of 18% if they used cooking fuels such as kerosene or liquid petroleum, and an increase of 11% when the kitchens did not have a ventilation system. For chronic cough, an increase in NO2 levels of 18% was found among those with chronic cough. The levels of NO2 among the examined 362 children were correlated with NO2 levels measured in their classrooms, all of which had opened windows so that the NO2 came from outdoors [62].

The effect of indoor nitrogen dioxide exposure on the incidence of respiratory symptoms and pulmonary function level was also examined in an American cohort of 1,567 Caucasian children aged between seven and eleven years and examined from 1983 through 1988 [63]. Nitrogen dioxide was assessed three indoor locations over 2 consecutive weeks in the winter and summer periods and household annual averages of the nitrogen dioxide concentrations were assessed as continuous variables and as four categories. Multiple logistic regression analysis of symptom reports after indoor monitoring then revealed that an increased cumulative incidence of lower respiratory symptoms was positively associated with a 15-ppb increase in the household annual nitrogen dioxide mean (odds ratio (OR) = 1.4, 95% confidence interval (95% Cl) 1.1 to 1.7). The response variable indicated the report of one or more of the following symptoms: chronic cough, chronic phlegm, attacks of shortness of breath with wheeze, chronic wheeze, or bronchitis. School girls showed a stronger association than boys (OR = 1.7, 95% Cl 1.3–2.2 vs. OR = 1.2, 95% Cl 0.9–1.5). An analysis of pulmonary function measurements showed no consistent effect of nitrogen dioxide. These results were consistent with earlier reports based on categorical indicators of household nitrogen dioxide sources and provided a specific association of respiratory diseases such as chronic cough with nitrogen dioxide as measured in children's homes [63].

Also, the SAPALDIA study examined the association between NO2 and chronic cough and it was reported that an increase of 10 μg/m^3 ^in pollutant levels was associated with an increase in the prevalence of chronic cough [64]. Next to this study, the Swiss Study on Childhood Allergy and Respiratory Symptoms with Respect to Air Pollution, Climate and Pollen (SCARPOL-study) [65] also reported that the symptom rate of chronic cough, adjusted for individual risk factors, was positively associated with NO2 [65].

Together, these data indicate that long-term NO2 exposure leads to increased incidences of chronic cough and decreased lung function. Also, weak associations between short-term NO2 exposure and respiratory symptoms and a decrement in lung function parameters were found in children, but not consistently in exposed women.

### Sulphur dioxide

With regard to one of the most dramatical urban environmental exposures to air pollutants, the London smogs [66-70], sulphur dioxide (SO2) is known as major respiratory irritant since many years. Next to its acute effects, SO2 may also be related to the incidence of chronic cough [12]. In total, 149 PubMed entries inclided the terms "cough" and "sulphur" (Fig. 3). Some of them, but others not, found associations between SO2 exposure and respiratory symptoms such as chronic cough and daily mortality and morbidity. In general, single-pollutant correlations sometimes disappeared when other pollutants such as suspended particulate matter (SPM) were included.

A study on indoor air pollution and respiratory health in urban and rural China revealed interesting relations between chronic cough and SO2 [71]: During the summer of 1999, data on the respiratory health outcomes and relevant covariates was collected from 3,709 Chinese adult individuals in the cities of Beijing, Anqing City, and in rural communities. Indoor SO2 was measured in a random sample of selected households and using logistic regression and controlling for important covariates (excluding PM10 and SO2) and familial intraclass correlation, highly significant differences were found between study areas in the prevalence of chronic cough. In general, the lowest prevalence of respiratory symptoms was observed in Anqing City, a higher prevalence in rural Anqing, and the highest prevalence in Beijing. However, median indoor concentrations of SO2 were similar in all three areas (Beijing: 14 microg/m3, Anqing City: 25 microg/m3, rural Anqing: 20 microg/m3) [71].

A further study in Canada also addressed the association of inhalable sulfates, SO2 and childhood chronic cough [72]: Preadolescent school children, aged 7–11 years, who resided in 10 rural Canadian communities areas of moderate and low exposure to regional sulfate and ozone pollution were examined. The communities were situated in central Saskatchewan, a low-exposure region, and in southwestern Ontario, an area with moderately increased SO2 exposures resulting from a long-distance atmospheric transport of polluted air masses. In this cross-sectional study, the annual mean and 90th percentile concentrations of inhalable sulfates were three times higher in Ontario than in Saskatchewan but levels of SO2 were low in both regions. After controlling for the effects of age, sex, parental smoking, parental education, and gas cooking, no significant regional differences were observed in rates of chronic cough [72]. In the SAPALDIA study, there were no data on the association between SO2 and chronic cough reported [64]. However, other European studies found an association between SO2 exposure and chronic cough [73].

### Ozone

Ozone is a very powerful oxidant and also a very toxic air pollutant [12,74]. As a gaseous air pollutant, its primary target organ is the respiratory tract [75] and exposure to even slightly elevated concentrations of ozone leads to a range of respiratory symptoms including chronic cough [76-78].

A variety of studies focused on the relation between ozone-exposure and chronic cough (Fig. 3) and revealed controversial results. For instance, the effects of ambient ozone (O3) on respiratory function and acute respiratory symptoms were assessed 7- to 9-yr-old schoolchildren followed longitudinally at 1- to 2-wk intervals over a period of 6 months at three schools in Mexico City. The maximum O3 level which was measured exceeded the World Health Organization guideline of 80 ppb and the U.S. standard of 120 ppb in every week. For an increase from lowest to highest in the mean O3 level during the 48 hr before spirometry (53 ppb), logistic regression estimated relative odds of 1.7 for a child reporting cough on the day of spirometry [79].

In the cross-sectional study in Canada with preadolescent school children, aged 7–11 years, in the low-exposure region central Saskatchewan, and in the moderately polluted area in southwestern Ontario, no significant regional differences were observed in rates of chronic cough. Here, the annual mean of the 1-hr daily maxima of ozone was higher in Ontario (46.3 ppb) than in Saskatchewan (34.1 ppb), with 90th percentile concentrations of 80 ppb in Ontario and 47 ppb in Saskatchewan and summertime 1-hr daily maxima means were 69.0 ppb in Ontario and 36.1 ppb in Saskatchewan [72].

In the Swiss SAPALDIA and SCARPOL studies, no associations between ozone and chronic cough were found [64,65].

In contrast to these epidemiological data, there is a large body of experimental evidence that ozone influences pulmonary vagal-sensory nerve fibers which are suggested to be major mediators of the cough reflex [24,80]. However, these effects may be reflected by the known short-term acute effects of ozone such as pulmonary function decrements, increased airway responsiveness and airway inflammation.

In this respect, exposure-response relations are non-linear for the respective associations between O3 and FEV1, inflammatory changes, and changes in hospital admissions, whereas it has been reported that the relation between percent change in symptom exacerbation among adults and asthmatics is linear. Also, the single-pollutant associations between ozone exposure and hospital admissions for respiratory diseases and daily mortality were shown to be statistically significant, even in multi-pollutant models.

### Other environmental causes

There is also a variety of other environmental factors which have been reported to contribute to the prevalence of chronic cough. In this respect, recent reports have demonstrated a relation between allergen such as Humicola fuscoatra in indoor air and chronic cough (combined with sputum eosinophilia) [81] or arsenic contaminated well water in Bangladesh [82]. Here, a prevalence comparison study of chronic cough and chronic bronchitis among subjects with or without arsenic exposure revealed a crude prevalence ratio for chronic cough and chronic bronchitis amounted to 2.1 (95% CI 0.7–6.1). The prevalence ratios for chronic cough increased with increasing exposure, i.e., 1.0, 1.6, 2.7 and 2.6 for the exposure categories using unexposed as the reference, indicating that long-term ingestion of arsenic exposure can cause chronic cough [82].

Also, depleted uranium, which is a radioactive heavy metal that is commonly used in missiles, has been shown to be associated with chronic cough as a health risk [83].

Next to microbially-related chronic cough [84], a further environmental cause may be found in exposure to Formaldehyde: [85]. A study on the relation of chronic respiratory symptoms and pulmonary function to indoor formaldehyde (HCHO) examined in a sample of 298 children (6–15 years of age) and 613 adults revealed an association to chronic cough.

## Conclusion

Environmental air pollution is not only one of the causes that might lead to severe diseases such as cancer or cardiorespiratory disease, but also associated with the prevalence of chronic cough both in adults and children.

Although episodes of incidental, dramatic air pollution are relatively rare, the currently found levels of exposure to pollutants especially in developing countries may increase the prevalence of chronic cough. Next to well-known air pollutants such as nitrogen dioxide, a number of other indoor and outdoor pollutants have recently been demonstrated to cause chronic cough and therefore, environmental factors have to be taken into account as potential initiators of both adult and pediatric chronic cough.

## Declaration of competing interests

The author(s) declare that they have no competing interests.

## Authors' contributions

BGK and AF have planed the study. BGK has peformed the data analysis and interpretation and drafted the article. TK, AVM and UW have all been involved in interpreting and discussing the data and revising the article critically for important intellectual content.
